# A perspective on the artificial intelligence’s transformative role in advancing diffractive optics

**DOI:** 10.1016/j.isci.2024.110270

**Published:** 2024-06-18

**Authors:** S.N. Khonina, N.L. Kazanskiy, A.R. Efimov, A.V. Nikonorov, I.V. Oseledets, R.V. Skidanov, M.A. Butt

**Affiliations:** 1 Samara National Research University, 443086 Samara, Russia; 2 Sber, Moscow, Russia; 3 Artificial Intelligence Research Institute (AIRI), Moscow, Russia; 4 Skolkovo Institute of Science and Technology (Skoltech), Moscow, Russia

**Keywords:** Applied sciences, Artificial intelligence

## Abstract

Artificial intelligence (AI) is transforming diffractive optics development through its advanced capabilities in design optimization, pattern generation, fabrication enhancement, performance forecasting, and customization. Utilizing AI algorithms like machine learning, generative models, and transformers, researchers can analyze extensive datasets to refine the design of diffractive optical elements (DOEs) tailored to specific applications and performance requirements. AI-driven pattern generation methods enable the creation of intricate and efficient optical structures that manipulate light with exceptional precision. Furthermore, AI optimizes manufacturing processes by fine-tuning fabrication parameters, resulting in higher quality and productivity. AI models also simulate diffractive optics behavior, accelerating design iterations and facilitating rapid prototyping. This integration of AI into diffractive optics holds tremendous potential to revolutionize optical technology applications across diverse sectors, spanning from imaging and sensing to telecommunications and beyond.

## Introduction

Diffractive optics, an integral subset of optics, focuses on manipulating light through diffraction patterns, marking its escalating importance in the contemporary world owing to its multifaceted applications across diverse domains.^1^ Leveraging the principles of interference and diffraction, diffractive optics facilitate the creation and production of intricate optical components that transcend the limitations of conventional refractive optics. Ranging from cutting-edge imaging apparatuses for medical diagnosis and astronomical observations to the development of high-definition displays and precision laser beam molding for telecommunications, diffractive optics emerges as a fundamental driver in advancing optical technology.^2^^,^^3^^,^^4^ Its capacity to downsize optical systems, augment resolution, and introduce inventive functionalities has spurred its widespread integration into consumer electronics, healthcare solutions, manufacturing processes, and scientific explorations, firmly establishing it as a cornerstone of contemporary optical engineering.

Despite the manifold benefits and applications of diffractive optics, researchers encounter various challenges in its utilization. Foremost among these is the intricate task of designing DOEs while maintaining precise control over diffraction patterns.^5^ This demands the application of sophisticated algorithms and optimization techniques, often coupled with advanced fabrication methods like lithography or laser writing.^6^ Furthermore, diffractive optics is susceptible to environmental factors such as temperature fluctuations and mechanical strains, which can induce undesired aberrations and compromise performance.^7^ Another obstacle arises from the limited spectral bandwidth of diffractive elements, constraining their effectiveness in broadband optical systems.^8^ Additionally, fabricating high-quality diffractive optics with large dimensions or high aspect ratios proves technically demanding and costly. Tackling these hurdles necessitates interdisciplinary collaboration and continuous research endeavors aimed at devising novel design approaches, materials, and fabrication methodologies to enhance the resilience and versatility of diffractive optical systems.

Free space optics (FSO) utilizes an array of optical components to transmit and receive optical signals through air or space, eliminating the necessity for physical cables. These components encompass lenses, mirrors, beam splitters, optical filters, spatial light modulators (SLMs), digital micromirror devices (DMDs), and DOEs. Lenses are pivotal for focusing and collimating light beams, while mirrors serve to steer and redirect optical signals. Beam splitters facilitate the division of light into multiple beams, catering to diverse applications like optical communication or sensing.^9^^,^^10^^,^^11^ Optical filters are employed to selectively transmit specific wavelengths of light while blocking others, aiding in signal processing and noise reduction. SLMs and DMDs provide dynamic control over the phase and amplitude of light waves, thereby enabling adaptive beamforming and holographic imaging.^12^^,^^13^ The use of AI tools with tunable diffractive devices^14^ seems particularly promising in the field of adaptive optics^15^ and aberration corrections.^16^ DOEs offer efficient beam shaping, steering, and focusing capabilities in compact and lightweight formats.^8^^,^^12^ Together, these optical elements constitute the foundation of FSO systems, empowering high-speed data transmission, remote sensing, and other vital applications in demanding environments.^17^

## The evolution of AI and algorithmic contributions to diffractive optics

The history of AI spans numerous decades, marked by continuous evolution and significant breakthroughs. Its roots can be traced back to the mid-20th century, notably with the pioneering contributions of Alan Turing, who conceptualized a universal machine capable of emulating human computation.^18^ The pivotal Dartmouth Conference in 1956 saw the coining of the term “artificial intelligence”, laying the groundwork for formalizing AI as a distinct field of study.^19^ Early AI systems primarily relied on rule-based methods and symbolic reasoning, exemplified by projects like the Logic Theorist developed by Allen Newell and Herbert Simon.^20^ Progress, however, faced obstacles due to computational limitations and data scarcity. The resurgence of interest in the 1980s, spurred by advancements in neural networks (NNs) and machine learning (ML) algorithms, revitalized AI research.^21^ Subsequent developments in expert systems, natural language processing, and robotics broadened the application spectrum of AI.^22^ In recent years, the convergence of big data, cloud computing, and deep learning (DL) has ushered in remarkable breakthroughs, revolutionizing industries across healthcare, finance, transportation, and entertainment.^23^ Today, AI technologies such as ML, natural language processing, and computer vision have become integral components of daily life, propelling innovations, and reshaping the landscape of human-machine interaction.^24^

AI has become a pivotal asset in propelling the field of diffractive optics forward. By harnessing AI algorithms and ML techniques, researchers can streamline the design and optimization processes of DOEs with unparalleled efficiency and accuracy.^25^^,^^26^^,^^27^ AI facilitates the exploration of extensive design spaces, facilitating the discovery of innovative DOEs with customized diffraction patterns tailored to specific performance criteria.^28^ Furthermore, AI-driven optimization algorithms not only augment the functionality and effectiveness of diffractive optics but also mitigate the necessity for extensive trial-and-error experimentation. Moreover, AI-based methodologies simplify the integration of diffractive optics into intricate optical systems, fostering seamless interoperability and heightened overall performance. Through the unification of AI and diffractive optics, researchers stand poised to unveil novel capabilities and applications, propelling the boundaries of optical engineering and heralding transformative advancements across diverse sectors, spanning from telecommunications and imaging to sensing and beyond.^29^^,^^30^ Nano-color routing has emerged as a highly popular and widely discussed topic in light field manipulation, image sensing, and DL integration.^31^ Traditional dye filters used in commercial applications have long faced limitations, including subpar signal-to-noise ratios, restricted optical efficiency, and miniaturization challenges.^32^ However, the advent of bandpass-free color routing has unlocked new possibilities, enabling remarkable optical spectral efficiency and sub-wavelength scale operations in image sensing applications. This innovation has brought about a paradigm shift, fundamentally transforming the field by offering a promising solution to overcome the constraints of traditional dye filters.^33^

**Figure 1:**
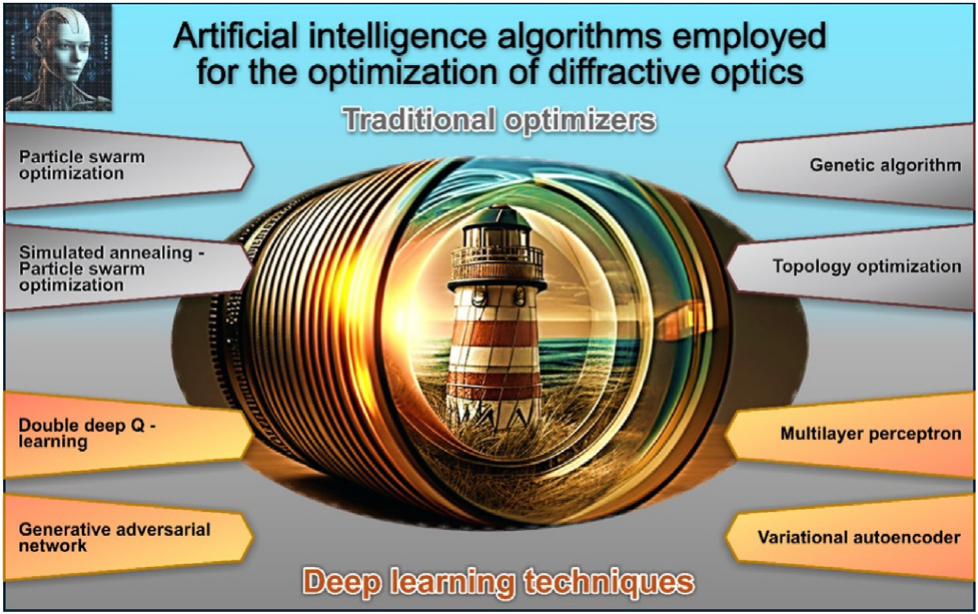
AI algorithms employed in the advancement of diffractive optics

In a recent study by Jia et al.,^34^ the inverse design of cascaded DOEs was significantly enhanced using ML. Specifically, a bidirectional focusing diffractive lens was investigated in a cascaded configuration utilizing the diffractive optical neural network (DONN) ML approach. This lens comprised two on-axially cascaded multi-level diffractive lenses, featuring concentric rings with equal widths and varying heights in each lens. The optimization process involved regulating the elevation of each concentric ring. Notably, the diffractive lens demonstrated different focal lengths denoted as f_+_ and f_−_ for forward (Z+) and backward (Z−) light propagation, respectively. Fabrication of the designed lens was accomplished using a two-photon polymerization 3D printing technique. Critically, the proposed strategy was polarization insensitive and compact, suggesting its potential application in future optical imaging systems. In Figure 1, we delineate the AI algorithms pivotal to the advancement of diffractive optics, a subject carefully examined within this paper.

The application of foundational AI models has been expanding across various industrial sectors,^35^ such as medicine, chemistry,^36^ and industrial design.^37^ These models typically leverage large-scale pretrained transformers to generate innovative solutions tailored to the needs of a particular industry. A similar methodology is applicable to the field of nanophotonics, where custom-trained GPT-like AI models can facilitate the inverse design process.^38^

In our opinion, one prominent category of AI-based optimization algorithms is evolutionary algorithms, such as genetic algorithms (GA) and particle swarm optimization (PSO), which mimic the process of natural selection to iteratively refine designs. These algorithms explore a diverse range of design possibilities, gradually improving solutions over successive generations.^39^ Customary phase retrieval algorithms like the Gerchberg–Saxton (GS) and Yang–Gu algorithms often yield suboptimal results due to their susceptibility to local extreme points.^40^^,^^41^ This results in poor uniformity and increased noise in the diffraction patterns. To address these limitations, the search for new design methods led to the proposal of a hybrid algorithm named SA–PSO, combining simulated annealing (SA) with PSO.^42^ This hybrid approach overcomes the drawbacks of random disturbances and local optima, leading to improved performance compared to traditional algorithms like GS.^43^ The simulation results demonstrate the effectiveness of the SA–PSO hybrid algorithm in enhancing the quality of holographic projection through more accurate and uniform diffractive optical element (DOE) designs.

Topology optimization algorithms have also emerged as powerful tools in the development of diffractive optics, revolutionizing the design process.^44^^,^^45^ These algorithms utilize mathematical techniques to determine the optimal distribution of material within a given design domain, thereby enabling the creation of complex and efficient DOEs.^44^ By iteratively adjusting the shape and distribution of features within the optical element, topology optimization algorithms can maximize performance metrics such as diffraction efficiency or spectral bandwidth while minimizing undesirable effects like optical aberrations. This approach allows for the creation of lightweight, compact, and highly customizable diffractive optical components tailored to specific applications, ranging from beam shaping and wavefront manipulation to imaging and sensing systems.^46^

Another type is gradient-based optimization methods, including gradient descent^47^ and stochastic gradient descent,^48^ which iteratively adjust parameters based on the gradient of a cost function to converge toward optimal solutions. Additionally, ML algorithms like NNs and DL models can be trained to predict optimal designs based on historical data, offering efficient and tailored solutions for specific applications. Recent advancements in DL have garnered significant attention, with its performance surpassing that of human experts in certain fields. This technology has brought about a revolution in AI and computer science, leading to substantial progress in these domains. Each of these AI-driven optimization algorithms brings unique strengths to the table, providing researchers with powerful tools to navigate complex design spaces and push the boundaries of diffractive optics.

DL techniques have significantly impacted the development of diffractive optics by offering innovative solutions to traditional challenges. Double Deep Q-Learning (DDQN) has been applied in optimizing DOEs by efficiently exploring complex design spaces and enhancing the performance of optical systems through reinforcement learning-based optimization.^49^ Generative adversarial networks (GANs) have been employed to generate novel designs for DOEs, facilitating the creation of highly customized and efficient optical components.^50^ Variational autoencoders (VAEs) have played a role in learning compact representations of diffractive optical structures, enabling efficient encoding and reconstruction of complex optical patterns.^51^ Additionally, multilayer perceptrons (MLPs) have been applied in modeling the behavior of diffractive elements and predicting their optical properties, leading to improved understanding and optimization of diffractive optics.^52^ These DL techniques offer novel approaches to design, simulate, and optimize diffractive optical systems, contributing to advancements in areas such as imaging, sensing, and telecommunications.

Recently, a DL model was employed for computer hologram generation.^53^ This model underwent training to approximate the inverse light propagation from a random intensity-only image. Consequently, the trained model exhibited the capability to produce phase-only DOEs from arbitrary images. Notably, the computational times of the trained network were reduced while maintaining performance levels comparable to those achieved by the GS algorithm. In another instance, a technique for creating DOEs from arbitrary datasets was proposed, utilizing a deep residual NN.^54^ Through training, the network learns to estimate the inverse light propagation process. Investigational outcomes indicate the model’s effectiveness in generating a DOE from a test image not included in the training set. Comparable approaches hold promise for enhancing the quality of digital holograms, computing computer-generated holograms, and designing other DOEs. Enhanced accomplishment can be attained by refining network architecture or employing progressive methods for scheming light propagation to create data conducive to NN generalization.

A significant challenge in simulating sub-wavelength diffractive optics, also identified as meta-optics, arises from their multi-scale characteristics.^55^ While individual scatterers, termed meta-atoms, necessitate full-wave electromagnetic simulations, modeling the entire meta-optical system can be accomplished through ray/Fourier optics. However, prevailing simulation methods typically employ the local phase approximation (LPA), disregarding inter-meta-atom couplings. In a recent study,^56^ researchers introduced a physics-informed NN in conjunction with the overlapping boundary method to efficiently model meta-optics while considering all meta-atom couplings. This technique’s effectiveness was demonstrated through the design of cylindrical meta-lenses with 1mm apertures, exhibiting superior efficiency compared to those designed under LPA. Experimental validation revealed a maximum intensity enhancement of up to 53% for the inverse-designed meta-lens. Moreover, this method enables the design of large aperture meta-optics (∼10^4^ − 10^5^λ) within a reasonable time frame (nearly 15 min on a GPU) without depend on on LPA.

The rapid progress of digital neural networks (DNNs) and the wide availability of large training datasets have enabled a diverse range of ML applications, including image analysis, speech recognition, and machine vision.^57^ However, as performance improves, models become more complex, leading to increased computational needs. This rise in NN utilization and complexity has resulted in higher energy consumption and challenges in real-time decision-making, especially in scenarios lacking significant computational resources. These issues are critical for machine vision in autonomous systems, where compactness, lightweight design, and low power consumption are essential for onboard processing, alongside minimal latency, high accuracy, and robust operation. Meeting these demands requires ongoing development of innovative hardware and software solutions as the requirements on machine vision systems continue to grow.

**Figure 2:**
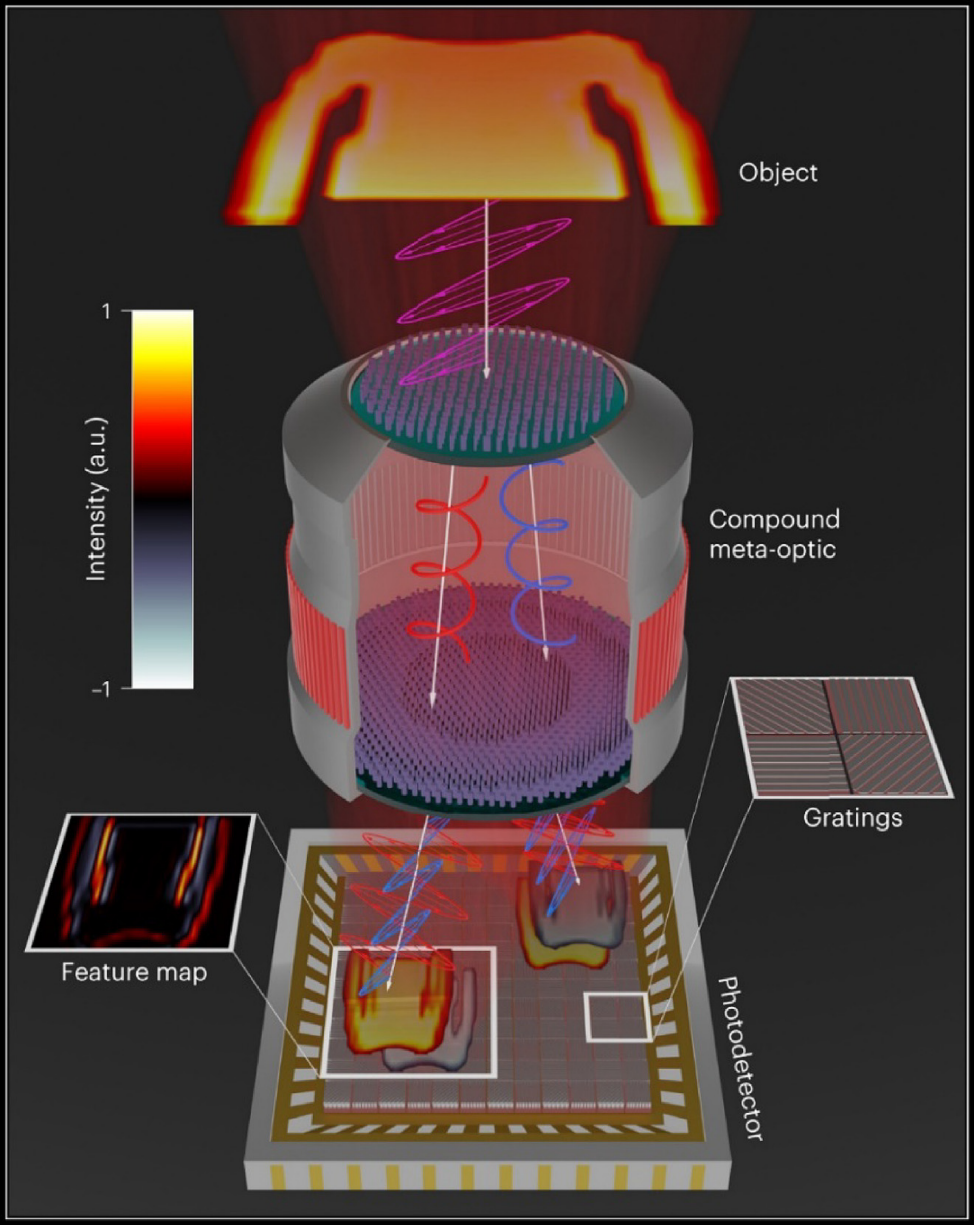
Meta-imager: It facilitates multichannel signal processing to substitute convolution operations within a DNN Employing a bilayer meta-optic system encoded with predetermined kernels, optical convolution is attained using an incoherent light source for object illumination. Positive and negative values are discerned and captured as feature maps through a polarization-sensitive photodetector. Each photodetector pixel is equipped with an oriented grating for sorting polarized signals. Reproduced with the permission.^58^

Zheng et al. proposed a meta-imager designed to collaborate with a digital back end for transferring computationally intensive convolution operations into high-speed, low-power optics.^58^ In this architecture, metasurfaces (MSs) facilitated both angle and polarization multiplexing, generating multiple information channels capable of conducting positive and negative convolution operations simultaneously (See Figure 2). This meta-imager was utilized for object classification, resulting in 98.6% accuracy in recognizing handwritten digits and 88.8% accuracy in identifying fashion images. Due to its compact design, rapid processing speed, and minimal power consumption, this approach holds potential for a broad array of applications in AI and machine vision.

**Figure 3:**
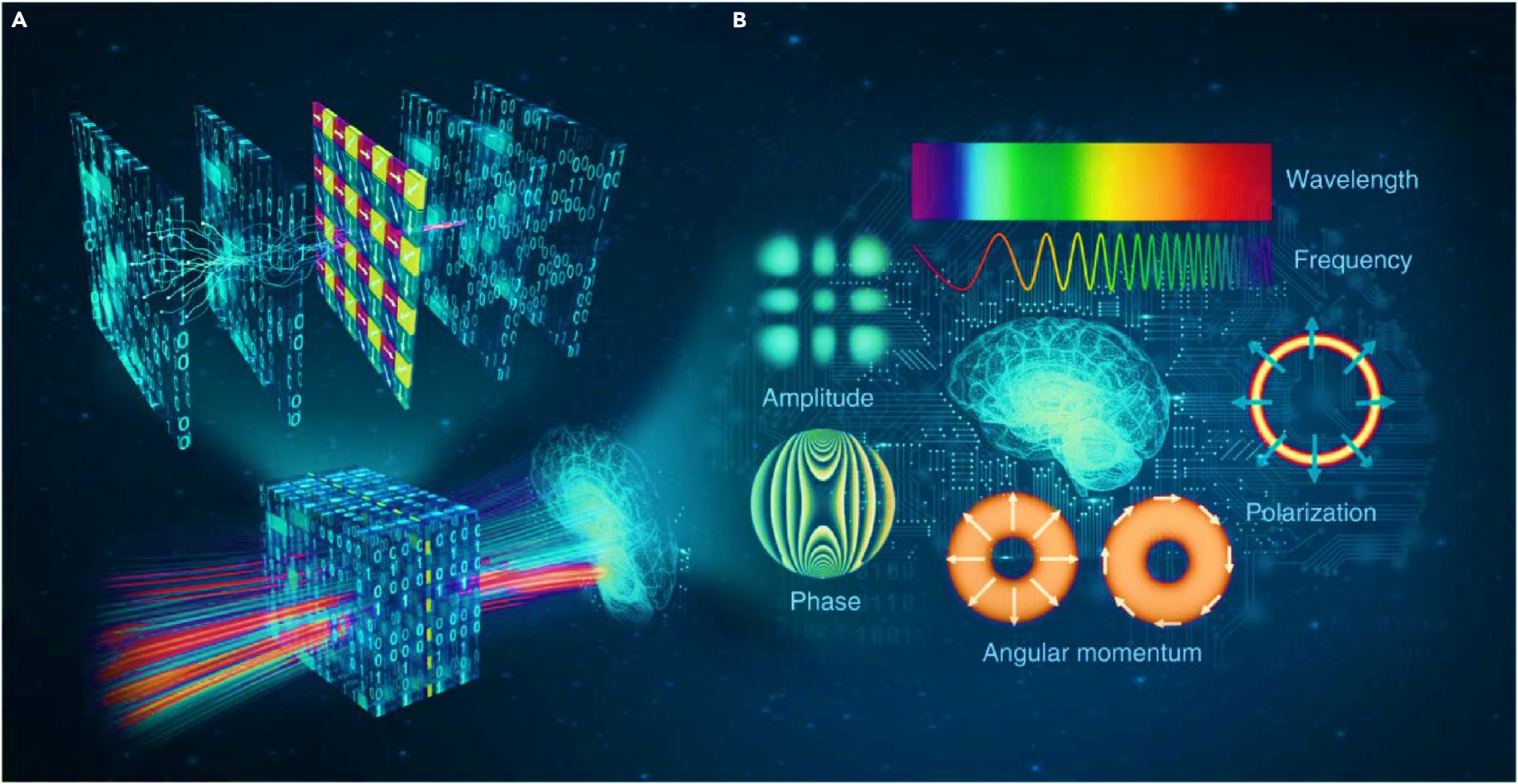
Diffractive neural network (A) Employing a sequence of structured diffractive surfaces and a basic polarizer array, polarization-multiplexed diffractive neural networks are employed. This setup facilitates communication between the trainable diffractive layers and the polarization elements within the diffractive volume, enabling a single network to generate multiple computing channels accessible through specific input and output polarization state combinations, (B) Harnessing the internal degrees of freedom of light opens up novel avenues for data multiplexing, thereby bolstering the performance and ability of optical diffractive networks.^64^

Recent advancements in ML have sparked considerable interest in optical computing research.^59^ One notable approach involves diffractive optical networks constructed from spatially engineered transmissive surfaces.^60^ These networks have been showcased for their ability to conduct all-optical statistical inference and execute arbitrary linear transformations through passive, free-space optical layers. Lately, there has been a demonstration that a diffractive NN could achieve an arbitrary complex-valued linear transformation between its input and output fields with minimal error, as long as the total number of engineered pixels in the network is adequate.^61^ In a broader context, a diffractive NN can be seen as a specialized optical system tailored for specific tasks, utilizing light as information carriers.^62^ The object field serves as a source of information flow, characterized by several fundamental properties that can be skillfully deployed to enhance the information processing capabilities of diffractive networks. Li et al. introduced polarization division multiplexing (PDM) to achieve multiple, arbitrarily selected linear transformations through a single diffractive network, a technique well-established in enhancing transmission capacity in telecommunications (Figure 3 (a)).^63^ Rather than relying on specialized materials sensitive to polarization, their polarization-multiplexed diffractive networks utilize standard diffractive surfaces with trainable coefficients independent of input light polarization. To enhance sensitivity to diverse polarization states and polarization mode diversity, a fixed linear polarizer array set at 0°, 45°, 90°, and 135° is integrated with trainable diffractive surfaces. This combination assigns distinct linear transformations to different input and output polarization states. Experimental demonstrations have shown that a single well-trained polarization-multiplexed diffractive network can perform multiple (2 or 4) specified linear transformations simultaneously—an achievement not yet realized with conventional materials or metamaterial-based designs. This polarization-multiplexed diffractive computing approach holds promise for developing passive, all-optical processors capable of parallel execution of multiple inference and optical information processing tasks.

The study presented by Li et al. was part of a broader effort to enhance the capacity of optical diffractive computing by leveraging light’s internal degrees of freedom, including polarization, spectrum, coherence, and orbital angular momentum, alongside spatial degrees of freedom (Figure 3 (b)). With this multidimensional upgrade, diffractive NNs can transmit optical signals across more autonomous channels, potentially enabling all-optical multiplexed diffractive processors capable of parallel task execution. Polarization multiplexing also offers the advantage of halving effective bandwidth compared to single-polarization transmission, facilitating the realization of high-dimensional diffractive NNs using lower numerical-aperture optics. This is crucial for plummeting the physical footprint of diffractive NNs and relaxing requirements on interlayer distances. Additionally, while current diffractive network designs typically assume monochromatic, spatially coherent, forward-propagating input fields, exploring computational imaging methods that utilize partial coherence and evanescent waves could enhance imaging performance, particularly spatial resolution, and could potentially be adapted to diffractive NNs. Considering light’s internal degrees of freedom as a source of information gain could lead to significant advancements in high-performance optical diffractive computing schemes.

**Figure 4:**
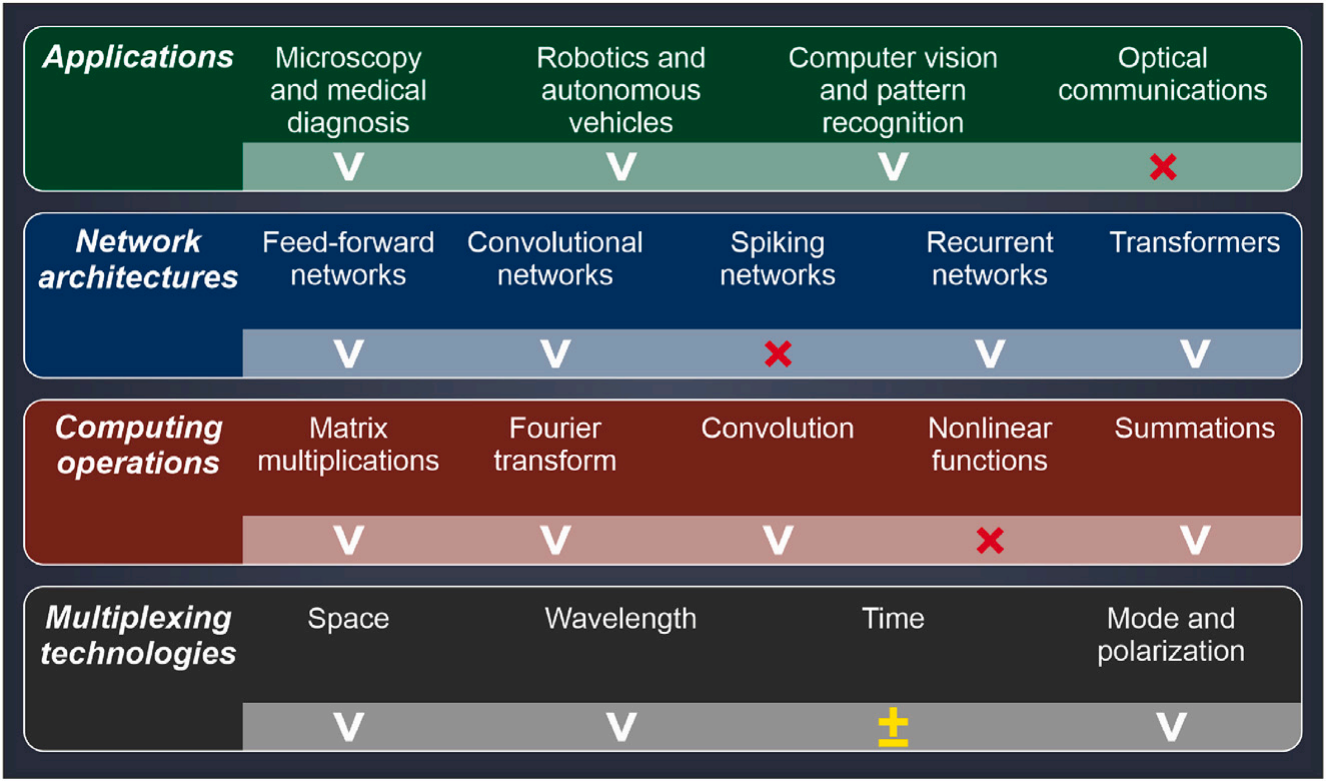
Potential applications and various properties of optical neural networks based on diffractive optics, compared to other photonic techniques such as Mach–Zehnder interferometers, microcombs, and waveguides

Creating non-linear optical systems remains a crucial technological hurdle, pivotal to the advancement of fully optical deep neural networks (DNNs).^65^ The inherent strength of optical computing, its ability to efficiently perform linear matrix operations, opens up intriguing prospects, such as substituting the computationally intensive linear components of DNNs with optical analogs.^66^ Among the most promising avenues is the application of optical approximations to replace the linear part of self-attention mechanisms within large transformer models. Pioneering studies in this domain,^67^ suggest that such optical substitutes for matrix multiplication could significantly decrease the computational costs, both during the inference and training phases of transformer models. A list of computing operators and multiplexing technologies^68^ supported by optical systems, along with the DNN architectures that can be augmented with these optical computational elements and their applications, is shown in Figure 4. We also evaluated the feasibility of using diffractive optics for each capability, indicating full support with a checkmark and partial support with +/−.

## Role of AI in the manufacturing of DOEs

The standard procedure for manufacturing DOEs typically involves a series of sequential steps, starting with the design phase where optical engineers manually create or modify the DOE layout based on theoretical models and computational simulations. Once the design is finalized, it undergoes fabrication using techniques such as lithography, etching, or direct writing methods to pattern the desired optical structures onto a substrate.^69^ This process often requires iterative adjustments and extensive trial and error to achieve the desired performance metrics. The selection of materials remains highly restricted for imprinting diffractive optics even today. Key concerns revolve around crucial process parameters such as curing time, viscosity, lifespan, particularly resistance to UV light or heat, and optical characteristics like refractive index and Abbe number.^3^

UV lithography has traditionally been extensively used to fabricate DOEs.^70^ This technique allows for writing entire structures simultaneously, but the minimum feature size is limited to approximately 1 μm. While many 1-D and 2-D periodic grating structures do not require extremely fine details, such precision is crucial for diffractive optics with circular structures. In these cases, the spacing between features typically decreases toward the edge of the element, resulting in features smaller than 1 μm. Similarly, 1-D structures like sub-wavelength gratings also necessitate smaller feature sizes throughout the structure. Advanced fabrication techniques, such as focused ion beam (FIB)^71^ and electron-beam lithography (EBL),^72^ are essential for creating these finer structures. Vijayakumar et al. presented the results of fabricating DOEs directly on a single-mode fiber tip using an FIB system (Nova Nanolab 600 from FEI).^71^ Writing DOEs directly on fiber tips is highly significant for applications such as endoscopy and optical trapping. The DOE transforms the laser beam to a phase and intensity profile that meets specific requirements. Because the DOE is located directly on the fiber, no additional alignment is necessary, resulting in a more compact system—an important feature for endoscopic applications.

With the continuous reduction in printable critical dimensions in photolithography, off-axis illumination (OAI) has emerged as a highly effective resolution-enhancement technique to tackle these challenges. Consequently, this has led to much stricter requirements, including the need for higher diffractive efficiency of the DOEs utilized in OAI systems. While advancements in design algorithms have optimized the phase profiles of DOEs, the fabrication process has become the primary constraint, resulting in energy losses. Tolerance analysis serves as a crucial method to assess fabrication accuracy requirements, particularly beneficial for deep UV applications with small structures and stringent tolerances. A subpixel DOE simulation model was employed for tolerance analysis by translating abstract fabrication structure errors into measurable subpixel phase matrices.^73^ This model enabled the investigation of four fabrication errors: misetch, misalignment, feature size error, and feature rounding error. Through simulation experiments, systematic studies of fabrication errors in five typical DOEs used in a 90-nm scanning photolithography illumination system were conducted. These findings are instrumental in refining high-precision DOE design algorithms and optimizing the fabrication process.^73^

Direct laser lithography stands out as a quintessential technique utilized in the manufacturing of DOEs. This method involves the precise utilization of laser technology to pattern intricate optical structures onto a substrate, ensuring unparalleled precision and detail in the fabrication process.^74^ Direct laser lithography offers advantages in terms of scalability, allowing to production of both small-scale prototypes and large-scale commercial batches with consistent quality. Additionally, it enables rapid prototyping and customization, empowering optical engineers to swiftly iterate designs and adapt to evolving requirements.^75^ Through direct laser lithography, manufacturers can achieve high-resolution DOEs with exceptional optical performance, making them a cornerstone in the realm of diffractive optics fabrication.^76^

Although additive manufacturing using multi-photon direct laser writing is now considered a major tool in fabricating future nano/micro-objects and optical components, it remains limited by the low throughput of the writing process.^77^^,^^78^ To address this issue, massively parallelizing the write process is a very promising avenue. However, simultaneous writing of structures in close spatial proximity generates fabrication artifacts, collectively referred to as “proximity effects”, which significantly limit the accessible structure resolution. Arnoux et al. systematically investigated the experimental parameters influencing these effects using specifically designed N×N spot DOEs.^79^ Computer simulations demonstrate that these effects can be modeled remarkably successfully by considering point spread function overlap and diffusion processes. The concept’s usefulness was illustrated by designing a parallel write approach to plot periodic structures with submicron inter-object distances, largely overcoming proximity effects.

Fast atom beam (FAB) etching is another advanced fabrication technique that employs a directed stream of neutral atoms accelerated to high velocities.^80^ This method offers precise control over etching depth and surface morphology, making it ideal for creating intricate nanostructures and complex patterns. FAB etching minimizes damage to the substrate and produces smooth, high-resolution features, making it particularly useful for fabricating DOE s and other micro/nanoscale devices with stringent design requirements. Lee et al. presented both scalar and vector analyses of sawtooth gratings with a period of 2.0 μm, utilizing Fourier transformation and rigorous coupled wave analysis (RCWA).^81^ Additionally, the fabrication of these gratings on a slanted silicon substrate using a novel fast atom beam (FAB) etching method was presented. Initially, the optical and geometrical properties of the sawtooth gratings were investigated and optimized to meet phase-matching requirements. The estimated 1st diffraction efficiencies for TM polarization and scalar approximation were 73% and 100%, respectively. Subsequently, the optimized sawtooth gratings, based on the two diffraction analysis methods, were successfully fabricated using the FAB etching method. Finally, through a hot-embossing process suitable for mass production, a 100 μm thick poly-methyl methacrylate (PMMA) material was replicated from the sawtooth-patterned silicon substrate. Optical testing revealed a 1st diffraction efficiency for TM polarization of 63.0% for the replicated PMMA.^81^

In contrast, AI-powered optimization techniques revolutionize this procedure by leveraging ML algorithms to automate and enhance various stages of DOEs development. These techniques employ sophisticated algorithms to explore vast design spaces, rapidly generating and evaluating numerous design iterations to identify optimal solutions efficiently. AI algorithms can adaptively learn from previous designs and performance data, guiding the optimization process toward achieving desired specifications with higher precision and faster convergence rates. By integrating AI into the optimization pipeline, manufacturers can significantly reduce development time and cost while improving the overall performance and manufacturability of DOEs.^82^ ABN Cleanroom Technology secures backing for the advancement of Artificial Cleanroom Intelligence (ACI). The $585,000 allocated for innovation support was specifically earmarked for advancing the development of a SmartBox designed to implement ACI technology in both established and newly constructed cleanroom facilities.^83^

## Take away message

AI technologies like DL have revolutionized sectors such as healthcare and finance, becoming integral components of daily life. Within the field of diffractive optics, AI algorithms and ML techniques are increasingly utilized to streamline design processes, optimize DOEs, and enhance overall performance. Moreover, various techniques and methods employed in the fabrication of DOEs are discussed in this paper. Traditional methods like UV lithography and advanced techniques such as focused ion beam (FIB) and electron-beam lithography (EBL) are widely used to fabricate DOEs. Additionally, emerging technologies like FAB etching and additive manufacturing are also assisting the realization of DOEs. The challenges associated with fabrication processes and the ongoing efforts to overcome them, including tolerance analysis and parallelization of fabrication are highlighted. The significance of direct laser lithography, with its unparalleled advantages in scalability and rapid prototyping, cannot be overstated.

We believe that the future potential of AI algorithms in advancing DOEs is highly promising. With the continuous evolution of AI, there lies the opportunity to transform the design, optimization, and manufacturing processes of DOEs. Through the utilization of ML and other AI methodologies, researchers can direct expansive design landscapes more efficiently, leading to the development of DOEs with unprecedented performance capabilities and versatile functionalities. AI algorithms play a crucial role in automating the generation of intricate DOE designs customized for specific applications like beam shaping, imaging, or optical encryption. Additionally, AI-powered optimization techniques can significantly improve the efficiency and precision of fabrication methods, facilitating the production of DOEs with heightened accuracy and faster turnaround times. Overall, the integration of AI into DOE advancement promises to unlock novel frontiers in optical engineering, enabling innovative applications across diverse sectors such as telecommunications, augmented reality, and biomedical imaging.
